# Supplementary material for: A brief measure of complete subjective well-being in Germany: A population-based validation of a German version of the Flourish Index (FI) and the Secure Flourish Index (SFI)
Source: PLoS One. 2023 Nov 28;18(11):e0284892. doi: 10.1371/journal.pone.0284892 (PMC10684093; doi:10.1371/journal.pone.0284892)
Supplement: S1 File — (DOCX) [file pone.0284892.s001.docx]

**Supporting Information**

**“A Brief Measure of Complete Subjective Well-Being in Germany: A Population-Based Validation of a German Version of the Flourish Index (FI) and the Secure Flourish Index (SFI)”**

#### S1 Table. Flourish Index (FI) and Secure Flourish Index (SFI) – correlations and descriptive statistics (*N_Study 1_*=192).

| **Measure** | **Statement/ Question** | **Pearson´s correlation coefficients** | | | | | | | | | | | | **Descriptives** | | | | | |
| --- | --- | --- | --- | --- | --- | --- | --- | --- | --- | --- | --- | --- | --- | --- | --- | --- | --- | --- | --- |
|  |  | D1.1 | D1.2 | D2.1 | D2.2 | D3.1 | D3.2 | D4.1 | D4.2 | D5.1 | D5.2 | D6.1 | D6.2 | ***M*** | ***SD*** | ***Min*** | ***Max*** | ***Skew*** | ***Kurt*** |
| FI | D1.1 | 1.000 |  |  |  |  |  |  |  |  |  |  |  | 7.21 | 2.02 | 0 | 10 | -1.18 | 1.24 |
| FI | D1.2 | 0.681^***^ | 1.000 |  |  |  |  |  |  |  |  |  |  | 7.04 | 1.80 | 2 | 10 | -0.81 | 0.28 |
| FI | D2.1 | 0.500^***^ | 0.602^***^ | 1.000 |  |  |  |  |  |  |  |  |  | 6.78 | 1.87 | 2 | 10 | -0.53 | -0.05 |
| FI | D2.2 | 0.572^***^ | 0.686^***^ | 0.608^***^ | 1.000 |  |  |  |  |  |  |  |  | 7.27 | 2.17 | 0 | 10 | -0.85 | 0.2 |
| FI | D3.1 | 0.469^***^ | 0.521^***^ | 0.313^***^ | 0.536^***^ | 1.000 |  |  |  |  |  |  |  | 6.95 | 2.34 | 0 | 10 | -0.76 | -0.04 |
| FI | D3.2 | 0.584^***^ | 0.553^***^ | 0.425^***^ | 0.554^***^ | 0.671^***^ | 1.000 |  |  |  |  |  |  | 6.42 | 2.72 | 0 | 10 | -0.86 | 0.32 |
| FI | D4.1 | 0.167^*^ | 0.221^**^ | 0.196^**^ | 0.153^*^ | 0.326^***^ | 0.446^***^ | 1.000 |  |  |  |  |  | 7.39 | 1.70 | 1 | 10 | -0.52 | 0.45 |
| FI | D4.2 | 0.185^*^ | 0.285^***^ | 0.177^*^ | 0.217^**^ | 0.271^***^ | 0.256^***^ | 0.262^***^ | 1.000 |  |  |  |  | 5.91 | 2.29 | 0 | 10 | -0.65 | 0.25 |
| FI | D5.1 | 0.505^***^ | 0.499^***^ | 0.316^***^ | 0.396^***^ | 0.529^***^ | 0.473^***^ | 0.254^***^ | 0.226^**^ | 1.000 |  |  |  | 7.24 | 2.18 | 0 | 10 | -0.93 | 0.48 |
| FI | D5.2 | 0.591^***^ | 0.593^***^ | 0.340^***^ | 0.450^***^ | 0.546^***^ | 0.522^***^ | 0.220^**^ | 0.210^**^ | 0.765^***^ | 1.000 |  |  | 6.74 | 2.61 | 0 | 10 | -0.78 | -0.2 |
| SFI | D6.1 | 0.452^***^ | 0.405^***^ | 0.425^***^ | 0.440^***^ | 0.228^**^ | 0.324^***^ | -0.009 | 0.211^**^ | 0.310^***^ | 0.320^***^ | 1.000 |  | 7.31 | 2.98 | 0 | 10 | -1.05 | -0.09 |
| SFI | D6.2 | 0.486^***^ | 0.412^***^ | 0.420^***^ | 0.444^***^ | 0.252^***^ | 0.373^***^ | 0.015 | 0.196^**^ | 0.288^***^ | 0.280^***^ | 0.890^***^ | 1.000 | 7.68 | 2.76 | 0 | 10 | -1.27 | 0.57 |

*Notes:* ^*^*p*<0.05, ^**^*p*<0.01, ^***^*p*<0.001; *M*=Mean; *SD*=Standard deviation; *Min*=Minimum; *Max*=Maximum; *Skew*=Skewness; *Kurt*=Excess kurtosis.

#### S2 Table. Flourish Index (FI) and Secure Flourish Index (SFI) – correlations and descriptive statistics (*N_Study 2_*=13,268).

| **Measure** | **Statement/ Question** | **Pearson´s correlation coefficients** | | | | | | | | | | | | | | **Descriptives** | | | | | |
| --- | --- | --- | --- | --- | --- | --- | --- | --- | --- | --- | --- | --- | --- | --- | --- | --- | --- | --- | --- | --- | --- |
|  |  | D1.1 | D1.2 | D2.1 | D2.2 | D3.1 | D3.2 | D4.1 | D4.2 | D5.1 | D5.2 | D6.1 | D6.2 | ***M*** | ***SD*** | | ***Min*** | ***Max*** | ***Skew*** | ***Kurt*** |  |
| FI | D1.1 | 1.000 |  |  |  |  |  |  |  |  |  |  |  | 6.98 | 2.08 | | 0 | 10 | -0.99 | 0.66 |  |
| FI | D1.2 | 0.766^***^ | 1.000 |  |  |  |  |  |  |  |  |  |  | 6.90 | 1.88 | | 0 | 10 | -0.97 | 0.78 |  |
| FI | D2.1 | 0.523^***^ | 0.496^***^ | 1.000 |  |  |  |  |  |  |  |  |  | 6.45 | 2.11 | | 0 | 10 | -0.7 | -0.04 |  |
| FI | D2.2 | 0.677^***^ | 0.701^***^ | 0.565^***^ | 1.000 |  |  |  |  |  |  |  |  | 7.07 | 2.27 | | 0 | 10 | -0.88 | 0.12 |  |
| FI | D3.1 | 0.577^***^ | 0.591^***^ | 0.418^***^ | 0.592^***^ | 1.000 |  |  |  |  |  |  |  | 6.75 | 2.64 | | 0 | 10 | -0.98 | 0.65 |  |
| FI | D3.2 | 0.496^***^ | 0.525^***^ | 0.328^***^ | 0.508^***^ | 0.677^***^ | 1.000 |  |  |  |  |  |  | 7.11 | 2.23 | | 0 | 10 | -0.86 | 0.02 |  |
| FI | D4.1 | 0.283^***^ | 0.311^***^ | 0.209^***^ | 0.278^***^ | 0.411^***^ | 0.419^***^ | 1.000 |  |  |  |  |  | 7.35 | 1.83 | | 0 | 10 | -0.8 | 0.97 |  |
| FI | D4.2 | 0.256^***^ | 0.261^***^ | 0.221^***^ | 0.271^***^ | 0.274^***^ | 0.279^***^ | 0.328^***^ | 1.000 |  |  |  |  | 7.11 | 2.19 | | 0 | 10 | -0.76 | 0.23 |  |
| FI | D5.1 | 0.529^***^ | 0.545^***^ | 0.314^***^ | 0.477^***^ | 0.445^***^ | 0.439^***^ | 0.313^***^ | 0.267^***^ | 1.000 |  |  |  | 7.33 | 2.28 | | 0 | 10 | -1.02 | 0.59 |  |
| FI | D5.2 | 0.557^***^ | 0.574^***^ | 0.331^***^ | 0.481^***^ | 0.453^***^ | 0.458^***^ | 0.291^***^ | 0.254^***^ | 0.829^***^ | 1.000 |  |  | 6.83 | 2.56 | | 0 | 10 | -0.88 | 0.07 |  |
| SFI | D6.1 | 0.365^***^ | 0.325^***^ | 0.288^***^ | 0.336^***^ | 0.296^***^ | 0.249^***^ | 0.118^***^ | 0.241^***^ | 0.227^***^ | 0.236^***^ | 1.000 |  | 7.69 | 2.67 | | 0 | 10 | -1.22 | 0.51 |  |
| SFI | D6.2 | 0.373^***^ | 0.340^***^ | 0.287^***^ | 0.348^***^ | 0.321^***^ | 0.269^***^ | 0.146^***^ | 0.213^***^ | 0.241^***^ | 0.247^***^ | 0.842^***^ | 1.000 | 8.03 | 2.53 | | 0 | 10 | -1.46 | 1.27 |  |

*Notes:* ^*^*p*<0.05, ^**^*p*<0.01, ^***^*p*<0.001; *M*=Mean; *SD*=Standard deviation; *Min*=Minimum; *Max*=Maximum; *Skew*=Skewness; *Kurt*=Excess kurtosis.

#### S3 Table. Flourish Index (FI) and Secure Flourish Index (SFI) – correlations and descriptive statistics (*N_Study 3_*=317).

| **Measure** | **Statement/ Question** | **Pearson´s correlation coefficients** | | | | | | | | | | | | **Descriptives** | | | | | | |  |
| --- | --- | --- | --- | --- | --- | --- | --- | --- | --- | --- | --- | --- | --- | --- | --- | --- | --- | --- | --- | --- | --- |
|  |  | D1.1 | D1.2 | D2.1 | D2.2 | D3.1 | D3.2 | D4.1 | D4.2 | D5.1 | D5.2 | D6.1 | D6.2 | | ***M*** | ***SD*** | ***Min*** | ***Max*** | ***Skew*** | ***Kurt*** | |
| FI | D1.1 | 1.000 |  |  |  |  |  |  |  |  |  |  |  | | 6.16 | 2.30 | 0 | 10 | -0.81 | 0.03 | |
| FI | D1.2 | 0.745^***^ | 1.000 |  |  |  |  |  |  |  |  |  |  | | 6.49 | 2.01 | 0 | 10 | -1.03 | 1.04 | |
| FI | D2.1 | 0.538^***^ | 0.530^***^ | 1.000 |  |  |  |  |  |  |  |  |  | | 6.36 | 2.21 | 0 | 10 | -0.75 | -0.01 | |
| FI | D2.2 | 0.610^***^ | 0.642^***^ | 0.575^***^ | 1.000 |  |  |  |  |  |  |  |  | | 6.62 | 2.35 | 0 | 10 | -0.82 | 0.14 | |
| FI | D3.1 | 0.514^***^ | 0.583^***^ | 0.326^***^ | 0.436^***^ | 1.000 |  |  |  |  |  |  |  | | 6.45 | 2.72 | 0 | 10 | -0.84 | -0.04 | |
| FI | D3.2 | 0.581^***^ | 0.656^***^ | 0.401^***^ | 0.483^***^ | 0.705^***^ | 1.000 |  |  |  |  |  |  | | 6.51 | 2.49 | 0 | 10 | -0.89 | 0.33 | |
| FI | D4.1 | 0.258^***^ | 0.374^***^ | 0.162^**^ | 0.204^***^ | 0.465^***^ | 0.445^***^ | 1.000 |  |  |  |  |  | | 7.15 | 1.88 | 0 | 10 | -0.68 | 0.7 | |
| FI | D4.2 | 0.322^***^ | 0.339^***^ | 0.177^**^ | 0.314^***^ | 0.301^***^ | 0.378^***^ | 0.278^***^ | 1.000 |  |  |  |  | | 7.09 | 2.11 | 0 | 10 | -0.59 | 0.06 | |
| FI | D5.1 | 0.492^***^ | 0.546^***^ | 0.326^***^ | 0.389^***^ | 0.477^***^ | 0.489^***^ | 0.384^***^ | 0.378^***^ | 1.000 |  |  |  | | 7.32 | 2.31 | 0 | 10 | -1.28 | 1.58 | |
| FI | D5.2 | 0.570^***^ | 0.596^***^ | 0.387^***^ | 0.408^***^ | 0.493^***^ | 0.553^***^ | 0.370^***^ | 0.418^***^ | 0.823^***^ | 1.000 |  |  | | 6.59 | 2.66 | 0 | 10 | -0.88 | 0.14 | |
| SFI | D6.1 | 0.290^***^ | 0.250^***^ | 0.245^***^ | 0.273^***^ | 0.166^**^ | 0.292^***^ | 0.053 | 0.131^*^ | 0.155^**^ | 0.194^***^ | 1.000 |  | | 6.11 | 3.23 | 0 | 10 | -0.44 | -1.03 | |
| SFI | D6.2 | 0.304^***^ | 0.289^***^ | 0.197^***^ | 0.271^***^ | 0.165^**^ | 0.297^***^ | 0.090 | 0.134^*^ | 0.203^***^ | 0.184^**^ | 0.890^***^ | 1.000 | | 6.32 | 3.26 | 0 | 10 | -0.52 | -0.96 | |

*Notes:* ^*^*p*<0.05, ^**^*p*<0.01, ^***^*p*<0.001; *M*=Mean; *SD*=Standard deviation; *Min*=Minimum; *Max*=Maximum; *Skew*=Skewness; *Kurt*=Excess kurtosis.

#### S4 Table. Flourish Index (FI) and Secure Flourish Index (SFI) – correlations, descriptive statistics, and reliability (*N_Study 3_*=317).

| **Measure** | **Domain** | **Pearson´s correlation coefficients** | | | | | | | | **Descriptives** | | | | | | ***Cronbach’s α*** |
| --- | --- | --- | --- | --- | --- | --- | --- | --- | --- | --- | --- | --- | --- | --- | --- | --- |
|  |  | D1 | D2 | D3 | D4 | D5 | D6 | FI_D1-5_ | SFI_D1-6_ | ***M*** | ***SD*** | ***Min*** | ***Max*** | ***Skew*** | ***Kurt*** |  |
| FI, SFI | D1. Happiness and Life Satisfaction | 1.000 |  |  |  |  |  |  |  | 6.32 | 2.01 | 0 | 10 | -0.90 | 0.64 | 0.85 |
| FI, SFI | D2. Mental and Physical Health | 0.701^***^ | 1.000 |  |  |  |  |  |  | 6.49 | 2.02 | 0 | 10 | -0.81 | 0.36 | 0.73 |
| FI, SFI | D3. Meaning and Purpose | 0.672^***^ | 0.502^***^ | 1.000 |  |  |  |  |  | 6.48 | 2.41 | 0 | 10 | -0.88 | 0.32 | 0.82 |
| FI, SFI | D4. Character and Virtue | 0.430^***^ | 0.306^***^ | 0.532^***^ | 1.000 |  |  |  |  | 7.12 | 1.60 | 2.50 | 10 | -0.23 | -0.41 | 0.43 |
| FI, SFI | D5. Close Social Relationships | 0.619^***^ | 0.448^***^ | 0.571^***^ | 0.509^***^ | 1.000 |  |  |  | 6.95 | 2.37 | 0 | 10 | -1.05 | 0.82 | 0.90 |
| SFI | D6. Financial and Material Stability | 0.313^***^ | 0.287^***^ | 0.253^***^ | 0.134^*^ | 0.199^***^ | 1.000 |  |  | 6.21 | 3.15 | 0 | 10 | -0.50 | -0.92 | 0.94 |
| FI | D1-D5 | 0.869^***^ | 0.748^***^ | 0.842^***^ | 0.672^***^ | 0.810^***^ | 0.302^***^ | 1.000 |  | 6.67 | 1.66 | 0.50 | 10 | -0.88 | 0.73 | 0.89 |
| SFI | D1-D6 | 0.843^***^ | 0.731^***^ | 0.801^***^ | 0.617^***^ | 0.755^***^ | 0.582^***^ | 0.951^***^ | 1.000 | 6.60 | 1.62 | 0.42 | 10 | -0.75 | 0.77 | 0.87 |

*Notes:* ^*^*p*<0.05, ^**^*p*<0.01, ^***^*p*<0.001; *M*=Mean; *SD*=Standard deviation; *Min*=Minimum; *Max*=Maximum; *Skew*=Skewness; *Kurt*=Excess kurtosis.

#### S5 Table. Descriptive statistics and reliability of the validation measures (*N_Study 3_*=317).

| **Measure** | ***M*** | ***SD*** | ***Min*** | ***Max*** | ***Cronbach’s α*** |
| --- | --- | --- | --- | --- | --- |
| Overall well-being | 6.99 | 1.39 | 2.27 | 10 | 0.92 |
| Accomplishment | 6.94 | 1.51 | 1.67 | 10 | 0.77 |
| Engagement | 7.08 | 1.45 | 2.33 | 10 | 0.64 |
| Positive emotions | 6.78 | 1.69 | 1 | 10 | 0.88 |
| Relationships | 7.21 | 1.97 | 1.33 | 10 | 0.85 |
| Meaning | 6.95 | 1.96 | 1 | 10 | 0.90 |
| Self-perceived health | 6.53 | 2.05 | 1 | 10 | 0.93 |
| Negative emotions | 4.24 | 1.73 | 1 | 9.33 | 0.73 |
| Loneliness | 4.40 | 2.70 | 1 | 10 | ― |
| Overall happiness | 7.08 | 1.94 | 1 | 10 | ― |

*Notes: M*=Mean; *SD*=Standard deviation; *Min*=Minimum; *Max*=Maximum.
