# Supplementary material for: A brief measure of complete subjective well-being in Germany: A population-based validation of a German version of the Flourish Index (FI) and the Secure Flourish Index (SFI)
Source: PLoS One. 2023 Nov 28;18(11):e0284892. doi: 10.1371/journal.pone.0284892 (PMC10684093; doi:10.1371/journal.pone.0284892)
Supplement: Attachment — Submitted filename: Response to Reviewers.docx [file pone.0284892.s002.docx]

**Response to Reviewers**

March 23, 2023

**RE: Resubmission of manuscript “A Brief Measure of Complete Subjective Well-Being in Germany: A Population-Based Validation of a German Version of the Flourish Index (FI) and the Secure Flourish Index (SFI)” (PONE-D-22-30338)**

**Dear Prof Richard Huan Xu and dear Reviewer,**

Thank you very much for taking the time and effort to read our manuscript “**A Brief Measure of Complete Subjective Well-Being in Germany: A Population-Based Validation of a German Version of the Flourish Index (FI) and the Secure Flourish Index (SFI)**”. We appreciate your positive feedback and constructive criticism.

In the following you will find our responses to your comments.

We hope that the revised manuscript is much improved and addresses the reviewer comments squarely and satisfactorily.

Please let us know if you have any questions.

Kind regards,

The authors

**Review Comments to the Authors and Responses by the Authors**

**Reviewer Comment 1:**

The manuscript aims to develop a German version of FI and SFI in a multi-stage translation and scale testing process on large sample analysis. Several indicators of reliability and validity were used to verify the measurement characteristics of FI and SFI in the German population. This manuscript is very valuable, especially in the field of measurement of subjective well-being. I am very pleased to review this manuscript. I have learned a lot from it. but there are several issues for the authors to consider:

**Our Response:**

Thank you very much for carefully reviewing our manuscript and for highlighting its significance for the field.

**Reviewer Comment 2:**

Abstract: In general, the end of the abstract should emphasize the significance of the manuscript's conclusions for research in the relevant field, which is obviously lacking in the current manuscript. In addition, the abstract should be brief, so the descriptions about sub-domains of FI and SFI should be removed. Furthermore, It is not recommended to write abbreviations such as "FI" and "FI" directly in the abstract, because abbreviations tend to confuse the reader. If authors insist on abbreviations, it is advisable to use the statement "The Flourish Index (FI) and the Secure Flourish Index (SFI)" when The Flourish Index and the Secure Flourish Index first appear in the abstract.

**Our Response:**

We followed your recommendation and spelled out the abbreviations prior to their first use. Given the possible length of 300 words, we decided to keep the information about the sub-domains because we consider them informative, but as requested, we added a conclusion statement: “Our translation of the FI and SFI, along with the empirical relationships that we found among the measures that we reviewed, will help scholars in Germany (and beyond) explore an expanded range of domains of well-being, including the comparatively neglected domains of character and virtue, physical health, and financial and material stability.” In addition, we added this sentence to our Conclusion, along with two references that debate the “neglected domains” and offer a more general critical dialog on flourishing.

**Reviewer Comment 3:**

Introduction: It’s OK. However, It would be better to add to the statement about the current state of psychological well-being measurement tools in Germany and the advantages of SI and SFI compared to them.

**Our Response:**

Thank you very much for this comment. Although such a comprehensive critical review of existing measures would be interesting, we think that this goes beyond the scope of this multi-study scale paper and may deserve a stand-alone paper (please also see our response to Comment 5b).

**Reviewer Comment 4:**

Study 1, 2, 3
a Please add inclusion and exclusion criteria for participants, and add the statement about how to ensure the data quality in Study 1, 2, 3.
b The Instruments section should have a reasonable reference source in Study 1, 2, 3.
c There are some table reference errors in line 244 and line 475. Table 3 seems redundant, please check it. Please check the whole paper for similar errors.
d In study 2, the reliability of validation measures, such as the Importance of health, etc., should be reported.
e In study 3, please explain why Pearson correlation analysis was used to verify the validity of convergence and what are the criteria for using this method. Moreover, in the Discussion, the convergence validity of FI and SFI was not reflected, and the correlation between variables was only simply introduced.

**Our Response:**

a) We are now more explicit about inclusion (for example, on the age range) and exclusion criteria (for example, missing data). Data quality was ensured through multiple ways:

All studies are based on nationwide samples and should therefore allow for a better generalizability of the results as compared to rather selective student samples. While Study 1 and 2 are based on a an offline-recruited nationwide random sample (multi-stage random process based on the ADM telephone master sample), Study 3 is based on a quota sample representative for gender, age, and federal state. The survey provider used for Study 3 ensures data quality of the sample through a double opt-in registration process, followed by an elaborate scoring and control process of panelists. Moreover, we provided monetary incentives to respondents completing the surveys.

In Study 1, we only present the FI/SFI, so we think that no further reference is needed. In Study 2, all instruments cite the relevant references. There are few exceptions, including the mental illness measure, which we created (thus, there is no reference) and we coded according to common practices (c.f., Siddiqui, N., Dwyer, M., Stankovich, J., Peterson, G., Greenfield, D., Si, L., & Kinsman, L. (2018). Hospital length of stay variation and comorbidity of mental illness: a retrospective study of five common chronic medical conditions. BMC health services research, 18, 1-10.) when constructing a binary indicator for any mental illness diagnosis (we provided more detail in the manuscript). Moreover, the one item measure for the importance of health measure was adapted from another instrument to which we now provide a reference. Finally, in Study 3, the PERMA profiler has been used in addition to the FI/SFI and a reference is provided.

b) Thank you very much for your careful reading. We corrected these errors and moved Table 3 to its correct position. We also reviewed the full paper and addressed any additional typographical errors.
c) Table 3 provides the requested information if applicable (some measures such as “importance of health” are single item measures while others are not scales such as the mental illness measure).
d) Similar to other studies (e.g., Keyes 1998), we used Pearson correlations for convergent validity analysis, for which we now provide references to support. We also provided guidance for classifying the strength of the coefficients including a reference.

**Reviewer Comment 5:**

Overall discussion
a In the section of Dimensional structure, the comparatively lower factor loading of D4-Character and Virtue D4.2 item should be discussed in more depth from the content of the item, instead of simply comparing the literature.
b Please add limitations and contributions to the literature of the manuscript at the end.

**Our Response:**

a) We thank the reviewer for this suggestion and have added the following text: “It is possible that the domain of Character and Virtue addresses a broader range of behaviors than the other domains. For example, the two items in our Happiness and Life Satisfaction domain (feeling happy and feeling satisfied) intuitively seem to go together, but as measured by the two items in our Character and Virtue domain, “promoting good” might in some cases might happen quite independent of “delaying happiness.” For example, one might find a great deal of happiness in promoting the good. There are no doubt other reasons why D4.2 might have lower factor loadings and given the relative newness of character measures in the flourishing literature—none of the other measures include this domain—this is clearly an area in need of further investigation.”
b) We thank the reviewer for this suggestion and agree that the contributions of the paper should be summarized and qualified by noting the limitations of this work. We have added the following text at the end of the Overall Discussion section: “*Limitations:* The present study has some limitations to note. Validation of the scale should include the assessment of the consistency or stability of the translated flourishing measures over time using test-retest reliability, which should be considered in subsequent studies. Additionally, some measures (such as household net income or diagnosed mental illnesses) might be subject to socially desirable responding what could distort our validity analysis. Furthermore, while we focused on the PERMA-Profiler to assess the convergent validity, we encourage future inquiry to also examine the relation of our translated FI and SFI measures with other existing instruments such as the “Fragebogen zur Lebenszufriedenheit” (FLZ) (51) or the German version of the Satisfaction with Life Scale SWLS) (52). With these limitations in mind, the findings from this study indicate that the German version of the FI and the SFI are appropriate measures for assessing distinct domains of well-being and overall flourishing in the German population.”
